# 4-Bromo-*N*,*N*′-bis­(4-methoxy­phen­yl)benzamidine

**DOI:** 10.1107/S1600536809040112

**Published:** 2009-10-17

**Authors:** Amlan K. Pal, Garry S. Hanan

**Affiliations:** a Département de Chimie, Université de Montréal, CP 6128, Succ. Centre-ville, Montréal, Québec, Canada H3C 3J7

## Abstract

The title compound, C_21_H_19_BrN_2_O_2_, is an amidine containing electron-donating meth­oxy groups and a bulky Br atom on the benzene rings. The solid-state structure reveals a non-centrosymmetric mol­ecule, with an *E* configuration around the C=N double bond. The C—N bonds show distinct amine [1.3689 (19) Å] and imine [1.285 (2) Å] characteristics. In the crystal, symmetry-related mol­ecules are linked *via* a very weak N—H⋯N inter­action, and C—H⋯O and C—H⋯π inter­actions.

## Related literature

For the use of benzamidine ligands as dimetallic tetra­midinate complexes, see: Chartrand & Hanan (2008 ▶). For structural features of this kind of benzamidine ligand, see: Alcock *et al.* (1988 ▶, 1994 ▶), Bortoluzzi *et al.* (2004 ▶), Barker *et al.* (1999 ▶). For structural features of acetamidine and formamidine ligands see: Norrestam *et al.* (1983 ▶); Cotton *et al.* (1997 ▶).
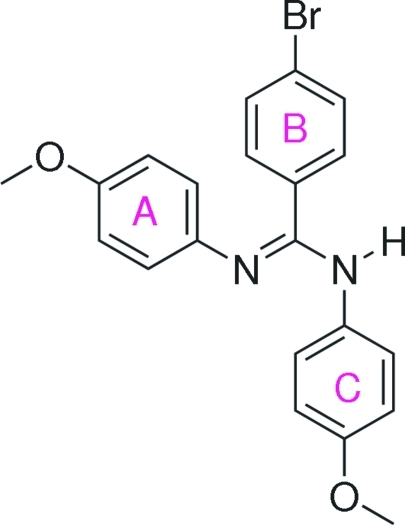


## Experimental

### 

#### Crystal data

C_21_H_19_BrN_2_O_2_
*M* *_r_* = 411.29
Orthorhombic, 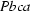

*a* = 9.2582 (6) Å
*b* = 16.8837 (10) Å
*c* = 23.9403 (14) Å
*V* = 3742.2 (4) Å^3^
*Z* = 8
Cu *K*α radiation
μ = 3.13 mm^−1^
*T* = 150 K
0.14 × 0.14 × 0.03 mm

#### Data collection

Bruker Microstar diffractometer
Absorption correction: multi-scan (*SADABS*; Bruker, 2001 ▶) *T*_min_ = 0.610, *T*_max_ = 0.910
52443 measured reflections
3396 independent reflections
3299 reflections with *I* > 2σ(*I*)
*R*_int_ = 0.062

#### Refinement

*R*[*F* ^2^ > 2σ(*F* ^2^)] = 0.029
*wR*(*F* ^2^) = 0.080
*S* = 1.07
3396 reflections
237 parameters
H-atom parameters constrained
Δρ_max_ = 0.33 e Å^−3^
Δρ_min_ = −0.59 e Å^−3^

### 

Data collection: *APEX2* (Bruker, 2007 ▶); cell refinement: *SAINT* (Bruker, 2007 ▶); data reduction: *SAINT*; program(s) used to solve structure: *SHELXS97* (Sheldrick, 2008 ▶); program(s) used to refine structure: *SHELXL97* (Sheldrick, 2008 ▶); molecular graphics: *SHELXTL* (Sheldrick, 2008 ▶); software used to prepare material for publication: *UdMX* (Maris, 2004 ▶).

## Supplementary Material

Crystal structure: contains datablocks I, global. DOI: 10.1107/S1600536809040112/su2139sup1.cif

Structure factors: contains datablocks I. DOI: 10.1107/S1600536809040112/su2139Isup2.hkl

Additional supplementary materials:  crystallographic information; 3D view; checkCIF report

## Figures and Tables

**Table 1:** Hydrogen-bond geometry (Å, °)

*D*—H⋯*A*	*D*—H	H⋯*A*	*D*⋯*A*	*D*—H⋯*A*
N1—H1⋯N2^i^	0.88	2.70	3.478 (2)	149
C14—H14*B*⋯O2^ii^	0.98	2.43	3.327 (2)	151
C7—H7⋯*Cg*2^iii^	0.95	2.67	3.3282 (18)	127
C13—H13⋯*Cg*3^iii^	0.95	2.69	3.6307 (17)	171

Symmetry codes: (i) 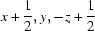; (ii) 
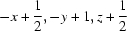
; (iii) 
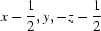
. *Cg*2 and *Cg*3 are the centroids of the C8–C13 and C15–C20 rings, respectively.

**Fig. 1.**
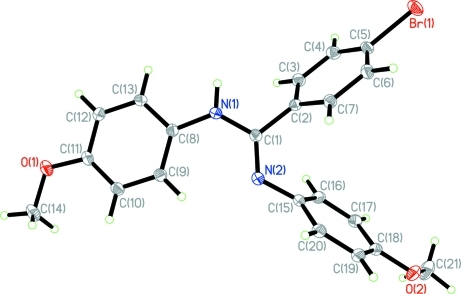
The molecular structure of the title compound, showing the numbering scheme and displacement ellipsoids drawn at the 50% probability level.

## supplementary crystallographic information

### Comment

Benzamidine ligands are of immense importance as amidinate complexes with dimetallic units (like Rh, Mo) can act as chromophores in light-harvesting devices (Chartrand & Hanan, 2008).

The title compound (Fig. 1) is an amidine containing electron donating methoxy groups on phenyl rings A and C, and a bulky bromine atom on phenyl ring B. Motives like these are of interest due to the fact that they can be incorporated into supramolecular assemblies via coordination chemistry. The C-N bonds show distinct amine [1.3689 (19) Å for C1-N1] and imine [1.285 (2) Å for C1═N2] characteristics, which on complexation become near equivalent bonds with a higher degree of delocalization. These values are similar to the values found in *N*,*N'*-diphenylbenzamidine [1.302 (7) Å and 1.360 (8) Å, respectively](Alcock *et al.*, 1988) and 4-methoxy-*N*,*N'*-diphenylbenzamidine [1.283 (2) Å and 1.372 (2) Å, respectively] (Bortoluzzi *et al.*, 2004), but differ to those found in *N*,*N'*-diphenylbenzamidinium nitrate [1.3266 (18) Å ] (Barker *et al.*, 1999).

It is already known that the difference between C-N and C═N depends on the degree of delocalisation in the N-C═N skeleton. In the title compound the difference is 0.0839 Å, whereas it is 0.058 Å in *N*,*N'*-diphenylbenzamidine (Alcock *et al.*, 1988), 0.046 Å in acetamidine (Norrestam and Mertz, 1983), and 0.06 Å in *N*,*N'*-di(*p*-tolyl)benzamidine (Alcock *et al.*, 1994). This correlation clearly proves that the degree of delocalisation depends on the substituents on the phenyl rings. In the title compound the bromine atom and the methoxy groups strongly influence the N-C═N congugation (0.0839 Å), in comparison with the unsubstituted compound *N*,*N'*-diphenylbenzamidine (0.058 Å).

From the torsion angles, C8-N1-C1-N2 = 13.9 (3)° and C8-N1-C1-C2 = 166.9 (14)°, it is revealed that the H-atom, H1, along with atom N1 and the phenyl substituent (ring A) at N2 are in an *E* (trans) configuration with respect to the C1═N2 bond. The solid state structure of the title compound indicates that the imine lone pair and the N1-H1 bond are on opposite sides of the molecule. This orientation hinders self association to give cyclic dimer formation, as observed in *N*,*N'*-di(p-chlorophenyl)formamidine (Cotton *et al.*, 1997). The widening of the N1-C1-N2 bond angle [121.89 (14)°] and the slight deviation from the ideal sp^2 ^bond angle (120°), also observed in *N*,*N'*-diphenylbenzamidine (120.4°) and *N*,*N'*-di(p-tolyl)benzamidine (120.8), is assumed to be due to intermolecular interactions.

In the crystal symmetry related molecules are linked by a very weak N-H···N, interaction and by C-H···O and C-H···π interactions (see Table 1 for details).

### Experimental

The title compound was prepared according to following procedure. A 250 ml round-bottomed flask was charged with *p*-bromobenzoic acid (10.0 g, 49.0 mmol) and SOCl_2_(45 ml). The resulting slurry was refluxed under a N_2_ atmosphere for 2 h to give a clear solution. The solution was cooled to rt and the unreacted SOCl_2_ was removed by distillation. The residue was then dried for 2 h under vacuum to give colourless crystalline *p*-bromobenzoyl chloride (11.0 g, 100%). Dry DCM (60 ml) and dry Et_3_N (20 ml, 143 mmol) were added to the residue at 283 K under a N_2_ atmosphere to give a brown precipitate. A solution of *p*-anisidine (7.0 g, 56 mmol) in another aliquot of dry DCM (40 ml) was then added to the reaction mixture over 30 min by syringe at 283 K to give a pale brown precipitate. The flask was then fitted with a reflux condenser and the slurry was heated to reflux for 10 h and a yellowish brown precipitate formed. After 10 h the mixture was cooled to rt and then evaporated to dryness to give a yellowish brown residue. Distilled water (200 ml) was then added and the resulting slurry was filtered and the solid rinsed with methanol (3 × 100 ml) to give colourless spongy crystalline 4-bromo-4'-methoxybenzanilide (15 g, 98%). (2) To a stirred solution of 4-bromo-4'-methoxybenzanilide (4.0 g, 13 mmol) in dry DCM (40 ml), in a 250 ml round-bottomed flask, was added a solution of PCl_5_ (5.0 g, 24 mmol) in dry DCM (30 ml) by a syringe at 283 K under a N_2_ atmosphere. The resulting slurry was then allowed to come to rt and was stirred for 2 h to give a clear bright yellow solution. After 2 h a solution of *p*-anisidine (4.8 g, 39 mmol) in another aliquot of dry DCM (30 ml) was added with stirring under a N_2_ atmosphere, maintaining the temperature at 283 K, and then the solution was allowed to reach rt. This solution was then stirred at rt for 1 h, giving pale yellow precipitate, and then was evaporated to dryness yielding a pale yellow residue, which was poured into a beaker containing basic aqueous KOH solution (200 ml, pH>12) to give an off-yellow residue. This residue was then filtered off and rinsed with water (5 × 100 ml) and dried under vacuum. Slow evaporation of an EtOAc solution of this pale-yellow solid gave pale-yellow crystals of the title compound (4.6 g, 85%). Colourless crystalline plates, suitable for X-ray analysis, were obtained by slow evaporation of a concentrated EtOAc solution of the title compound.

^1^H NMR (DMSO-*d*_6_,400 MHz, 330 K): d 8.97 (b, s, 1H), 7.76 (b, s, 2H), 7.52 (d, *J *= 8 Hz, 2H), 7.21 (d, *J *= 8 Hz, 2H), 6.85 (b, s, 2H), 6.62 (b, s, 2H), 6.49 (b, s, 2H), 3.66 (d, *J *= 37 Hz, 6H) p.p.m. Elemental analysis: expected for C_21_H_19_N_2_O_2_Br; C = 61.33%, H = 4.66%, N = 6.81%; found: C = 61.04%, H = 4.57%, N = 6.75%.

### Refinement

The H-atoms were included in calculated positions and treated as riding atoms: amine N—H 0.88 Å, aromatic C—H 0.95 Å, methyl C—H 0.98 Å, with U_iso_(H) = k × U_eq_(parent N- or C-atom), where k = 1.2 for the amine and aromatic H-atoms and 1.5 for the methyl H-atoms.

### Crystal data

**Table:**

C_21_H_19_BrN_2_O_2_	*F*(000) = 1680
*M**_r_* = 411.29	*D*_x_ = 1.460 Mg m^−^^3^
Orthorhombic, *P**b**c**a*	Cu *K*α radiation, λ = 1.54178 Å
Hall symbol: -P 2ac 2ab	Cell parameters from 36728 reflections
*a* = 9.2582 (6) Å	θ = 4.8–67.9°
*b* = 16.8837 (10) Å	µ = 3.13 mm^−^^1^
*c* = 23.9403 (14) Å	*T* = 150 K
*V* = 3742.2 (4) Å^3^	Plate, colorless
*Z* = 8	0.14 × 0.14 × 0.03 mm

### Data collection

**Table:**

Bruker Microstar diffractometer	3396 independent reflections
Radiation source: Rotating Anode	3299 reflections with *I* > 2σ(*I*)
Helios optics	*R*_int_ = 0.062
Detector resolution: 8.3 pixels mm^-1^	θ_max_ = 68.1°, θ_min_ = 5.2°
ω scans	*h* = −11→9
Absorption correction: multi-scan (*SADABS*; Bruker, 2001)	*k* = −19→20
*T*_min_ = 0.610, *T*_max_ = 0.910	*l* = −28→28
52443 measured reflections	

### Refinement

**Table:**

Refinement on *F*^2^	Primary atom site location: structure-invariant direct methods
Least-squares matrix: full	Secondary atom site location: difference Fourier map
*R*[*F*^2^ > 2σ(*F*^2^)] = 0.029	Hydrogen site location: inferred from neighbouring sites
*wR*(*F*^2^) = 0.080	H-atom parameters constrained
*S* = 1.07	*w* = 1/[σ^2^(*F*_o_^2^) + (0.0478*P*)^2^ + 1.6131*P*] where *P* = (*F*_o_^2^ + 2*F*_c_^2^)/3
3396 reflections	(Δ/σ)_max_ = 0.001
237 parameters	Δρ_max_ = 0.33 e Å^−^^3^
0 restraints	Δρ_min_ = −0.59 e Å^−^^3^

### Special details

**Table:**

Experimental. X-ray crystallographic data for I were collected from a single-crystal sample, which was mounted on a loop fiber. Data were collected using a Bruker microstar diffractometer equiped with a Platinum 135 CCD Detector, a Helios optics and a Kappa goniometer. The crystal-to-detector distance was 4.0 cm, and the data collection was carried out in 512 *x* 512 pixel mode. The initial unit-cell parameters were determined by a least-squares fit of the angular setting of strong reflections, collected by a 10.0 degree scan in 33 frames over three different parts of the reciprocal space (99 frames total).
Geometry. All e.s.d.'s (except the e.s.d. in the dihedral angle between two l.s. planes) are estimated using the full covariance matrix. The cell e.s.d.'s are taken into account individually in the estimation of e.s.d.'s in distances, angles and torsion angles; correlations between e.s.d.'s in cell parameters are only used when they are defined by crystal symmetry. An approximate (isotropic) treatment of cell e.s.d.'s is used for estimating e.s.d.'s involving l.s. planes.
Refinement. Refinement of *F*^2^ against ALL reflections. The weighted *R*-factor *wR* and goodness of fit *S* are based on *F*^2^, conventional *R*-factors *R* are based on *F*, with *F* set to zero for negative *F*^2^. The threshold expression of *F*^2^ > σ(*F*^2^) is used only for calculating *R*-factors(gt) *etc*. and is not relevant to the choice of reflections for refinement. *R*-factors based on *F*^2^ are statistically about twice as large as those based on *F*, and *R*- factors based on ALL data will be even larger.

### Fractional atomic coordinates and isotropic or equivalent isotropic displacement parameters (Å^2^)

**Table:**

	*x*	*y*	*z*	*U*_iso_*/*U*_eq_	
Br1	1.11149 (2)	0.899798 (11)	0.150666 (8)	0.03899 (10)	
O1	0.40209 (14)	0.43856 (8)	0.43426 (5)	0.0390 (3)	
O2	0.40707 (14)	0.73586 (8)	−0.00500 (5)	0.0394 (3)	
N1	0.72340 (15)	0.60992 (8)	0.27898 (5)	0.0276 (3)	
H1	0.8063	0.6259	0.2930	0.033*	
N2	0.59343 (16)	0.60447 (8)	0.19620 (6)	0.0306 (3)	
C1	0.69482 (16)	0.63517 (9)	0.22578 (6)	0.0252 (3)	
C2	0.79459 (16)	0.69987 (9)	0.20695 (6)	0.0248 (3)	
C3	0.80061 (17)	0.77121 (9)	0.23594 (6)	0.0296 (3)	
H3	0.7407	0.7789	0.2677	0.035*	
C4	0.89304 (17)	0.83133 (10)	0.21905 (7)	0.0318 (4)	
H4	0.8963	0.8802	0.2387	0.038*	
C5	0.98037 (17)	0.81875 (9)	0.17299 (7)	0.0285 (3)	
C6	0.97609 (18)	0.74872 (10)	0.14314 (7)	0.0297 (3)	
H6	1.0365	0.7412	0.1115	0.036*	
C7	0.88162 (17)	0.68942 (10)	0.16033 (7)	0.0269 (3)	
H7	0.8767	0.6412	0.1399	0.032*	
C8	0.63946 (17)	0.56199 (9)	0.31467 (6)	0.0245 (3)	
C9	0.52398 (17)	0.51543 (9)	0.29732 (6)	0.0266 (3)	
H9	0.5004	0.5125	0.2588	0.032*	
C10	0.44259 (18)	0.47305 (9)	0.33623 (7)	0.0288 (3)	
H10	0.3637	0.4415	0.3240	0.035*	
C11	0.47627 (17)	0.47678 (9)	0.39259 (7)	0.0284 (3)	
C12	0.59362 (18)	0.52215 (10)	0.41022 (7)	0.0292 (3)	
H12	0.6182	0.5242	0.4487	0.035*	
C13	0.67413 (17)	0.56419 (9)	0.37168 (7)	0.0270 (3)	
H13	0.7540	0.5950	0.3840	0.032*	
C14	0.2813 (2)	0.39151 (11)	0.41797 (9)	0.0407 (4)	
H14A	0.2095	0.4251	0.3994	0.061*	
H14B	0.2379	0.3671	0.4511	0.061*	
H14C	0.3133	0.3500	0.3922	0.061*	
C15	0.55222 (17)	0.64111 (10)	0.14555 (6)	0.0274 (3)	
C16	0.49751 (18)	0.71759 (10)	0.14424 (6)	0.0295 (3)	
H16	0.4956	0.7479	0.1777	0.035*	
C17	0.44532 (19)	0.75103 (10)	0.09498 (6)	0.0309 (4)	
H17	0.4066	0.8031	0.0951	0.037*	
C18	0.45031 (17)	0.70775 (10)	0.04598 (6)	0.0305 (3)	
C19	0.50326 (18)	0.63055 (10)	0.04669 (6)	0.0313 (4)	
H19	0.5054	0.6005	0.0132	0.038*	
C20	0.55266 (19)	0.59746 (9)	0.09586 (7)	0.0305 (4)	
H20	0.5873	0.5445	0.0960	0.037*	
C21	0.3394 (3)	0.81124 (14)	−0.00580 (8)	0.0532 (5)	
H21A	0.2566	0.8110	0.0197	0.080*	
H21B	0.3061	0.8230	−0.0438	0.080*	
H21C	0.4086	0.8518	0.0061	0.080*	

### Atomic displacement parameters (Å^2^)

**Table:**

	*U*^11^	*U*^22^	*U*^33^	*U*^12^	*U*^13^	*U*^23^
Br1	0.03179 (15)	0.02995 (14)	0.05522 (16)	−0.00738 (7)	0.00078 (7)	0.01143 (7)
O1	0.0405 (7)	0.0416 (7)	0.0348 (6)	−0.0134 (5)	0.0058 (5)	0.0085 (5)
O2	0.0449 (7)	0.0458 (7)	0.0275 (6)	−0.0014 (6)	−0.0100 (5)	0.0050 (5)
N1	0.0228 (7)	0.0332 (7)	0.0268 (6)	−0.0063 (5)	−0.0028 (5)	0.0050 (5)
N2	0.0293 (7)	0.0358 (8)	0.0268 (7)	−0.0084 (5)	−0.0012 (6)	0.0049 (5)
C1	0.0225 (7)	0.0276 (8)	0.0256 (7)	−0.0012 (6)	0.0017 (6)	0.0018 (6)
C2	0.0210 (7)	0.0272 (7)	0.0262 (7)	−0.0007 (6)	−0.0033 (6)	0.0036 (6)
C3	0.0278 (8)	0.0323 (8)	0.0287 (7)	−0.0015 (7)	0.0017 (6)	−0.0016 (6)
C4	0.0329 (9)	0.0270 (8)	0.0357 (9)	−0.0027 (6)	−0.0027 (6)	−0.0023 (7)
C5	0.0232 (7)	0.0259 (8)	0.0365 (8)	−0.0026 (6)	−0.0039 (6)	0.0082 (6)
C6	0.0267 (8)	0.0315 (8)	0.0310 (8)	0.0013 (7)	0.0038 (6)	0.0056 (6)
C7	0.0266 (8)	0.0256 (8)	0.0284 (7)	0.0010 (6)	−0.0004 (6)	0.0022 (6)
C8	0.0223 (7)	0.0245 (7)	0.0266 (7)	0.0008 (6)	0.0010 (6)	0.0026 (6)
C9	0.0277 (8)	0.0240 (7)	0.0282 (7)	−0.0002 (6)	−0.0012 (6)	0.0000 (6)
C10	0.0283 (8)	0.0232 (7)	0.0349 (8)	−0.0033 (6)	−0.0013 (7)	0.0006 (6)
C11	0.0285 (8)	0.0250 (7)	0.0318 (8)	−0.0015 (6)	0.0050 (6)	0.0051 (6)
C12	0.0297 (8)	0.0321 (8)	0.0257 (7)	−0.0012 (6)	−0.0015 (6)	0.0032 (6)
C13	0.0222 (8)	0.0291 (8)	0.0297 (8)	−0.0020 (6)	−0.0026 (6)	0.0018 (6)
C14	0.0315 (10)	0.0351 (9)	0.0555 (11)	−0.0073 (7)	0.0091 (8)	0.0081 (8)
C15	0.0218 (8)	0.0353 (9)	0.0252 (7)	−0.0078 (7)	−0.0001 (6)	0.0042 (6)
C16	0.0259 (8)	0.0357 (9)	0.0268 (7)	−0.0033 (7)	−0.0001 (6)	−0.0035 (6)
C17	0.0268 (9)	0.0339 (9)	0.0319 (8)	−0.0009 (6)	−0.0015 (6)	0.0011 (7)
C18	0.0244 (8)	0.0397 (9)	0.0275 (8)	−0.0064 (7)	−0.0026 (6)	0.0044 (6)
C19	0.0313 (8)	0.0372 (9)	0.0254 (7)	−0.0053 (7)	0.0001 (6)	−0.0028 (6)
C20	0.0280 (9)	0.0319 (9)	0.0316 (8)	−0.0036 (6)	0.0007 (7)	−0.0002 (6)
C21	0.0551 (12)	0.0662 (14)	0.0383 (10)	0.0196 (11)	−0.0079 (9)	0.0126 (9)

### Geometric parameters (Å, °)

**Table:**

Br1—C5	1.9057 (15)	C9—H9	0.95
O1—C11	1.3723 (19)	C10—C11	1.386 (2)
O1—C14	1.426 (2)	C10—H10	0.95
O2—C18	1.369 (2)	C11—C12	1.395 (2)
O2—C21	1.419 (3)	C12—C13	1.382 (2)
N1—C1	1.3689 (19)	C12—H12	0.95
N1—C8	1.410 (2)	C13—H13	0.95
N1—H1	0.88	C14—H14a	0.98
N2—C1	1.285 (2)	C14—H14b	0.98
N2—C15	1.414 (2)	C14—H14c	0.98
C1—C2	1.500 (2)	C15—C16	1.387 (3)
C2—C7	1.388 (2)	C15—C20	1.399 (2)
C2—C3	1.391 (2)	C16—C17	1.394 (2)
C3—C4	1.388 (2)	C16—H16	0.95
C3—H3	0.95	C17—C18	1.383 (2)
C4—C5	1.384 (2)	C17—H17	0.95
C4—H4	0.95	C18—C19	1.393 (2)
C5—C6	1.382 (2)	C19—C20	1.381 (2)
C6—C7	1.392 (2)	C19—H19	0.95
C6—H6	0.95	C20—H20	0.95
C7—H7	0.95	C21—H21a	0.98
C8—C9	1.390 (2)	C21—H21b	0.98
C8—C13	1.403 (2)	C21—H21c	0.98
C9—C10	1.396 (2)		
			
C11—O1—C14	117.11 (14)	C10—C11—C12	119.65 (14)
C18—O2—C21	116.88 (14)	C13—C12—C11	120.01 (15)
C1—N1—C8	129.49 (13)	C13—C12—H12	120
C1—N1—H1	115.3	C11—C12—H12	120
C8—N1—H1	115.3	C12—C13—C8	120.83 (14)
C1—N2—C15	119.55 (14)	C12—C13—H13	119.6
N2—C1—N1	121.89 (14)	C8—C13—H13	119.6
N2—C1—C2	125.31 (14)	O1—C14—H14A	109.5
N1—C1—C2	112.80 (13)	O1—C14—H14B	109.5
C7—C2—C3	119.20 (14)	H14A—C14—H14B	109.5
C7—C2—C1	120.45 (14)	O1—C14—H14C	109.5
C3—C2—C1	120.35 (14)	H14A—C14—H14C	109.5
C4—C3—C2	120.83 (15)	H14B—C14—H14C	109.5
C4—C3—H3	119.6	C16—C15—C20	118.17 (14)
C2—C3—H3	119.6	C16—C15—N2	121.68 (14)
C5—C4—C3	118.70 (15)	C20—C15—N2	119.86 (16)
C5—C4—H4	120.6	C15—C16—C17	121.51 (15)
C3—C4—H4	120.6	C15—C16—H16	119.2
C6—C5—C4	121.78 (15)	C17—C16—H16	119.2
C6—C5—BR1	119.18 (12)	C18—C17—C16	119.47 (16)
C4—C5—BR1	119.04 (12)	C18—C17—H17	120.3
C5—C6—C7	118.73 (15)	C16—C17—H17	120.3
C5—C6—H6	120.6	O2—C18—C17	124.27 (16)
C7—C6—H6	120.6	O2—C18—C19	115.98 (15)
C2—C7—C6	120.74 (15)	C17—C18—C19	119.74 (15)
C2—C7—H7	119.6	C20—C19—C18	120.37 (15)
C6—C7—H7	119.6	C20—C19—H19	119.8
C9—C8—C13	118.79 (14)	C18—C19—H19	119.8
C9—C8—N1	124.55 (14)	C19—C20—C15	120.70 (15)
C13—C8—N1	116.63 (14)	C19—C20—H20	119.7
C8—C9—C10	120.38 (14)	C15—C20—H20	119.7
C8—C9—H9	119.8	O2—C21—H21A	109.5
C10—C9—H9	119.8	O2—C21—H21B	109.5
C11—C10—C9	120.32 (15)	H21A—C21—H21B	109.5
C11—C10—H10	119.8	O2—C21—H21C	109.5
C9—C10—H10	119.8	H21A—C21—H21C	109.5
O1—C11—C10	125.00 (15)	H21B—C21—H21C	109.5
O1—C11—C12	115.35 (14)		
			
C15—N2—C1—N1	169.37 (15)	C14—O1—C11—C10	0.0 (2)
C15—N2—C1—C2	−11.6 (2)	C14—O1—C11—C12	180.00 (15)
C8—N1—C1—N2	−13.9 (3)	C9—C10—C11—O1	178.93 (15)
C8—N1—C1—C2	166.90 (14)	C9—C10—C11—C12	−1.1 (2)
N2—C1—C2—C7	−58.9 (2)	O1—C11—C12—C13	−178.87 (15)
N1—C1—C2—C7	120.22 (16)	C10—C11—C12—C13	1.1 (2)
N2—C1—C2—C3	120.87 (18)	C11—C12—C13—C8	0.0 (2)
N1—C1—C2—C3	−59.99 (19)	C9—C8—C13—C12	−1.2 (2)
C7—C2—C3—C4	−0.5 (2)	N1—C8—C13—C12	176.93 (15)
C1—C2—C3—C4	179.68 (14)	C1—N2—C15—C16	−61.3 (2)
C2—C3—C4—C5	−0.6 (2)	C1—N2—C15—C20	125.04 (17)
C3—C4—C5—C6	1.0 (2)	C20—C15—C16—C17	−0.6 (2)
C3—C4—C5—BR1	−178.30 (12)	N2—C15—C16—C17	−174.34 (15)
C4—C5—C6—C7	−0.3 (2)	C15—C16—C17—C18	−1.2 (3)
BR1—C5—C6—C7	178.98 (12)	C21—O2—C18—C17	−6.8 (2)
C3—C2—C7—C6	1.2 (2)	C21—O2—C18—C19	173.81 (17)
C1—C2—C7—C6	−178.98 (14)	C16—C17—C18—O2	−177.47 (15)
C5—C6—C7—C2	−0.8 (2)	C16—C17—C18—C19	1.9 (2)
C1—N1—C8—C9	16.0 (3)	O2—C18—C19—C20	178.51 (15)
C1—N1—C8—C13	−162.04 (15)	C17—C18—C19—C20	−0.9 (2)
C13—C8—C9—C10	1.3 (2)	C18—C19—C20—C15	−0.9 (3)
N1—C8—C9—C10	−176.71 (15)	C16—C15—C20—C19	1.6 (2)
C8—C9—C10—C11	−0.2 (2)	N2—C15—C20—C19	175.47 (15)

### Hydrogen-bond geometry (Å, °)

**Table:**

*D*—H···*A*	*D*—H	H···*A*	*D*···*A*	*D*—H···*A*
N1—H1···N2^i^	0.88	2.70	3.478 (2)	149
C14—H14B···O2^ii^	0.98	2.43	3.327 (2)	151
C7—H7···Cg2^iii^	0.95	2.67	3.3282 (18)	127
C13—H13···Cg3^iii^	0.95	2.69	3.6307 (17)	171

Symmetry codes: (i) *x*+1/2, *y*, −*z*+1/2; (ii) −*x*+1/2, −*y*+1, *z*+1/2; (iii) *x*−1/2, *y*, −*z*−1/2.
